# What sampling device is the most appropriate for vaginal vault cytology in gynaecological cancer follow up?

**DOI:** 10.2478/v10019-012-0047-6

**Published:** 2012-11-09

**Authors:** Del Pup Lino, Canzonieri Vincenzo, Serraino Diego, Campagnutta Elio

**Affiliations:** 1 Gynaecology Oncology Department; 2 Pathology Department and; 3 Epidemiology Unit, National Cancer Institute CRO, Aviano, PN, Italy

Radiol Oncol 2012; 46(2): 166–169.

doi:10.2478/v10019-012-0019-x

The first and family names of the authors were not quoted adequately. The correct author’s first and given names are: Lino Del Pup, Vincenzo Canzonieri, Diego Serraino, Elio Campagnutta.

